# Primary Leiomyosarcoma of Breast in an Adolescent Girl: A Case Report and Review of the Literature

**DOI:** 10.1155/2012/491984

**Published:** 2012-03-22

**Authors:** Swapnil Ulhas Rane, Charu Batra, Uma Nahar Saikia

**Affiliations:** Department of Histopathology, Postgraduate Institute of Medical Education and Research, Chandigarh 160012, India

## Abstract

Leiomyosarcoma of the breast is a rare neoplasm, primarily reported in older women. Only 44 cases have been reported in world literature and to the best of our knowledge, no case has been reported from India till date. We report a case of primary breast leiomyosarcoma in an adolescent girl who underwent a lumpectomy for rapidly increasing lump in the left breast. Here we report the histological findings and immunohistochemical profile of this entity, along with a review of existing literature.

## 1. Introduction

Primary sarcomas of the breast are rare tumors accounting for less than 1% of all breast neoplasms, just a handful of which are leiomyosarcoma. This tumor occurs usually in postmenopausal women, with most of the reported cases being between the age of fifty and eighty years [1–35]. Its occurrence in very young girls [8, 20] is extremely rare and may be clinically mistaken for fibroadenoma. In this paper, we present the clinical features of an adolescent girl with primary leiomyosarcoma of the breast, its pathological features, and an up-to-date review of literature on the topic.

## 2. Case Report

### 2.1. Clinical Presentation and Examination

 A 19-year-old adolescent girl presented with a rapidly increasing, painless mass in the left breast for 6-month duration. On clinical examination, the mass measured 8 cm in diameter, was well defined, lobulated, firm, and mobile with the overlying skin and nipple-areola being normal. No axillary lymph nodes were palpable. The patient did not have any family history of breast cancer or any other comorbidity. An ultrasound examination of the breast identified the mass to be well circumscribed, oval and was diagnosed as likely to be a fibroadenoma. Systemic physical, radiological, and ultrasound examination did not identify any suspicious mass in any other part of the body. The patient underwent an excision of the breast lump with the aim of diagnosis and relief of symptoms.

### 2.2. Pathological Findings

**Figure 1:**
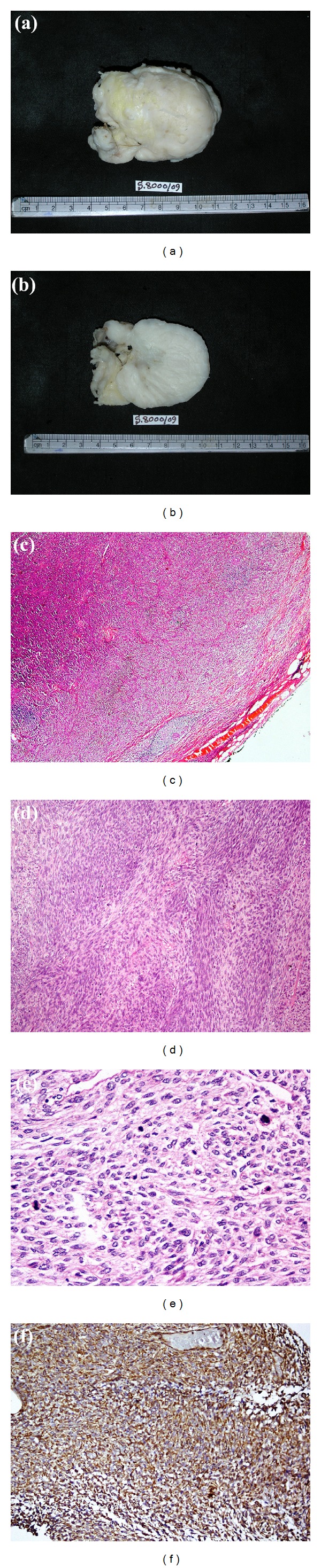
Gross photographs showing the external aspect (a) and cut surface (b) of the specimen showing a well-circumscribed and encapsulated mass with a thin rim of breast parenchyma surrounding it. The tumor is homogenous, whitish with areas of myxoid change. (c, d, e) Progressive increasing magnification of histology (40x, 100x and 400x) showing the well-encapsulated mass composed of intersecting fascicles of spindle cells with frequent mitoses. (f) Peroxidase-based immunohistochemistry for smooth muscle actin (SMA) showing diffuse, strong cytoplasmic positivity.

Grossly, the specimen composed of single, large, globular, and well-encapsulated mass measuring 7 cm in diameter (Figures 1(a), 1(b)). The mass was pearly white in color on both the outer surface as well as the cut surface with areas of whorling. No areas of hemorrhage, cystic degeneration, or necrosis were noted grossly. However, focal areas of myxoid change were seen. Microscopically, (Figures 1(c), 1(d), and 1(e)) the tumor was well-circumscribed, well-encapsulated, and composed of spindle cells arranged as intersecting long fascicles in a collagenous background. Individual tumor cells were moderately pleomorphic with round to oval nuclei, vesicular chromatin, and moderate amount of eosinophilic spindled cytoplasm. Binucleation and multinucleation were frequently noted, as was mitotic activity (20–25/10 high power field). Few myxoid areas were noted with interspersed thin-walled blood vessels and microscopic areas of necrosis. No epithelial component was noted in any part of the tumor. The mass was completely excised with a rim of normal breast tissue containing terminal duct lobular units surrounded by mild fibrosis.

Immunohistochemistry (Figure 1(f)) performed by the peroxidase technique showed the tumor cells to be strongly positive for smooth muscle actin and vimentin, while they were negative for pan-cytokeratin and desmin.

## 3. Discussion

Breast sarcomas are rare tumors accounting for about 0.5–1.0% of all breast neoplasms. Of these, cystosarcoma phylloides is the most common neoplasm, while only a handful of cases have been reported in the literature to be primary leiomyosarcoma of breast [1–35]. A comparison of clinicopathological features of primary leiomyosarcoma of breast reported in the English literature till date is presented in Table 1. In the largest series on breast sarcomas from the Mayo clinic, spread over a span of 90 years (1910–2000), Adem et al. [1] reported twenty five cases of primary breast sarcomas, of which only two were leiomyosarcoma. In the largest series on primary breast sarcomas from India, none of the 19 cases reported was a leiomyosarcoma [41]. Most of the patients reported till date of primary breast leiomyosarcoma have been postmenopausal, typically in the six-eighth decade. However, our patient is one of the two reported cases [8] of a young girl in her late teens to be diagnosed with a primary breast sarcoma. As with other sarcomas, prior chemotherapy for either a primary breast carcinoma or any other malignancy is a risk factor reported in the literature [6]. The exact cell of origin of this tumor is still debated with origin from smooth muscle of blood vessels, or that of the nipple areola complex and myofibroblasts undergoing myoid transformation being candidate histogenetic mechanisms [3, 25].

There is no clear consensus on the best treatment modality. However, the basic aim of treatment should be a complete excision with negative margins. Most cases reported have undergone mastectomy; however cases treated by lumpectomy have been reported albeit with a marginally higher incidence of recurrence and metastases [2, 4]. Prognosis is determined primarily by the adequacy of surgical resection. Although, there is no definite consensus on the use of adjuvant chemotherapy or radiotherapy, most patients reported till date have done well without any chemotherapy or radiotherapy, at least in the initial few years (see Table 1). The benefit of chemotherapy or radiation in preventing a recurrence many years later needs to be balanced by the risk of second malignancy. Most patients undergo mastectomy or at least wide local excision, as in our case. There is probably no role for axillary dissection, as there is no reason to believe that leiomyosarcomas follow a lymphatic route of dissemination. Even in cases which had palpable axillary nodes, axillary node dissection did not show any evidence of metastasis (Table 1). 

In conclusion, leiomyosarcoma of the breast is a rare entity with patients typically being in the 5th−7th decade; however it can rarely occur in younger patients as in our case. Morphologically it can be suspected by the typical histological features of circumscription, high cellularity and being composed of fusiform spindle cells having blunt end nuclei. Confirmation by an immunohistochemical profile of smooth muscle actin, vimentin, and desmin positivity is helpful; however, cases negative for some of these immunostains especially desmin have been reported. Demonstration of myofilaments on electron microscopy can help in those cases. 

## Figures and Tables

**Table 1:** Comparison of clinicopathological variables of all the cases of primary leiomyosarcoma of breast reported in the English literature.

Author	Year	Age/Sex	size (cm)	Mitosis (/10hpf)	Treatment	Ct/Rt	Final followup
Haagensen [35]	1971	77/F	8	very frequent	SM	—	Alive, 14 years
Pardo Mindan et al. [25]	1974	49/F	7	16	SM	—	Alive, 6 months
Barnes and Pietruszka [36]	1977	55/F	3	10	SM	—	Died 4 years 4 months later with basilar arterythrombosis
Hernandez [13]	1978	53/M	4	15	MRM	—	Alive, 1 year 2 months
Chen et al. [4]	1981	59/F	5.6	3	SM	—	Alive, 15 years
Callery et al. [37]	1984	56/F	2		SM	—	Alive, 39 months
Callery et al. [37]	1984	54/F	3		SM	—	Alive, 53 months
Yatsuka et al. [38]	1984	56/F	1.5	21	RM	—	Alive, 4 years 7 months
Gobardhan [9]	1984	50/F	9	5	MRM	—	Alive, 2 years
Nielsen [24]	1984	24/F	1.5 (1962) 1 (1965) 2 (1966)	2, 8, 14	WLE (1962), SM (1965)	—	Died 20 years later
Yamashina [33]	1987	62/F	2.5	11	SM	—	Alive, 2 years 2 months
Arista-Nasr et al. [2]	1989	50/F	4.5 (1980), 2.3 (1986)	4	WLE	—	Alive, 6 years 4 months
Parham et al. [26]	1992	52/F	3	29	SM	—	Alive, 6 months
Lonsdale and Widdison [21]	1992	60/F	2, 4 (18 mths later)	10	SM	—	Alive, 3 months,
Waterworth et al. [34]	1992	58/F	4	10	WLE + AC	—	Alive, 1 year
Wei et al. [16]	1993	36/F	4		MRM	—	Died 14 months later
Boscaino et al. [39]	1994	56/F	2.5/4	2	WLE (1981)/RM (1984)	—	Alive, 9 years
Boscaino et al. [39]	1994	45/F	1.9 (1985)/2.2 (1989)	2	E (1985)/WLE(1989)	—	Alive, 40 months, post wide local excision
Levy et al. [19]	1995	35/F	4	2	SM	—	Alive, 6 months
Falconieri et al. [7]	1997	83/F	6	20	RM	—	Alive, 10 months
Falconieri et al. [7]	1997	86/F	8	11	SM	—	Alive, 8 months
Ugras et al. [31]	1997	47/F	2	3	SM	—	Alive, 1 year 6 months
González-Palacios [10]	1998	62/F	3	10	SM	—	Alive, 17 years
Gupta et al. [12]	2000	80/F	6.5	5–8	SM + AC	—	Alive, 2 years
Székely et al. [30]	2001	73/F	4.8	20–22	SM	—	Alive, 1 year
Kusama et al. [17]	2002	55/F	0.5/-	few	WLE (1996, 1997)/SM (1998)	—	Alive, 4 years 8 months
Shinto et al. [28]	2002	59/F	12	19	SM	Ct	Alive, 8 months
Wei et al. [16]	2003	52/F	4	22	WLE	—	Alive, 3 months
Markaki et al. [22]	2003	42/F	14	50	MRM	Ct	Alive, 3 years
Markaki et al. [22]	2003	65/F	5,2	10	E	—	Alive, 18 months
Liang et al. [20]	2003	25/F	4	5	E	—	Alive, 32 months
Adem et al. [1]	2004	67/F	2		E	—	Died 7 months later
Adem et al. [1]	2004	55/F	4		SM	—	Died 77 months later
Jayaram et al. [15]	2004	55/F	12		MRM	—	Local recurrence
Lee et al. [18]	2004	44/F	3	6–12	SM	—	Alive, 13 months
Lee et al. [18]	2004	52/F	4.5	6–12	SM	—	Alive, 17 months
Stafyla et al. [29]	2004	53/F	23		MRM	Rt	Alive, 2 years
Munitiz et al. [23]	2004	58/F	4	14	MRM	—	Alive, 1 year
Gupta [11]	2006	37/F	8	15	WLE	—	Alive, 36 months
Vu et al. [32]	2006	-/F	23		SM	—	Alive, 10 months
De la Pena and Wapnir [6]	2008	50/F	3.2		SM	—	Alive, 11 months
Wong et al. [40]	2008	52/F	1.5	7	SM	—	Alive, 4 days
Cobanoglu et al. [5]	2009	64/F	3.5	12	MRM	—	Alive, 22 months
Fujita et al. [8]	2010	18/F	7.2	10	SM	Rt	Alive, 5 years
Present Case	2011	19/F	7	20–25	WLE	—	Alive, 3years

Ct: Chemotherapy, Rt: Radiotherapy, SM: Simple Mastectomy, RM: Radical Mastectomy, MRM: Modified Radical Mastectomy.
